# Perceived causes of adverse pregnancy outcomes and remedies adopted by Kalenjin women in rural Kenya

**DOI:** 10.1186/s12884-018-2041-5

**Published:** 2018-10-19

**Authors:** Roselyter Monchari Riang’a, Anne Kisaka Nangulu, Jacqueline E. W. Broerse

**Affiliations:** 1 School of Arts and Social Sciences, Moi University, P.O. Box 3900-30100, Eldoret, Kenya; 2 Athena Institute, Faculty of Science, Vrije Universiteit Amsterdam, De Boelelaan 1085, 1081 HV Amsterdam, The Netherlands; 3 Commission for University Education, Red Hill Road, off Limuru Road, Gigiri, PO Box 54999 – 00200, Nairobi, Kenya

**Keywords:** Pregnancy, Perceptions, Care-seeking behaviour, Maternal health, Kalenjin, Uasin-Gishu, Kenya

## Abstract

**Background:**

There have been few studies about the basis on which women in developing regions evaluate and choose traditional rather than western maternal care. This qualitative study explores the socio-cultural perceptions of complications associated with pregnancy and childbirth and how these perceptions influence maternal health and care-seeking behaviours in Kenya.

**Methods:**

Kalenjin women (*n* = 42) aged 18–45 years, who were pregnant or had given birth within the last 12 months, were interviewed. A semi-structured interview guide was used for data collection. A further nine key informant interviews with Traditional Birth Attendants (TBAs) who were also herbalists (*n* = 6), community health workers (CHWs) (*n* = 3) and a Maternal and Child Health (MCH) nursing officer (*n* = 1) were conducted. The data were analysed using MAXQDA12 software and categorised, thematised and analysed based on the symbolic dimensions of Helman’s (2000) ill-health causation aetiologies model.

**Results:**

Pregnancy complications are perceived as the consequence of pregnant women not observing culturally restricted and recommended behaviour during pregnancy, including diet; physical activities; evil social relations and spirits of the dead. These complications are considered to be preventable by following a restricted and recommended diet, and avoiding heavy duties, funerals, killing of animals and eating meat of animal carcasses, as well as restricting geographical mobility, and use of herbal remedies to counter evil and prevent complications.

**Conclusion:**

Delay in deciding to seek maternal care is a result of women’s failure to recognise symptoms and maternal health problems as potential hospital cases, and this failure stems from culturally informed perceptions of symptoms of maternal morbidity and pregnancy complications that differ significantly from biomedical interpretations. Some of the cultural maternal care and remedies adopted to prevent pregnancy complications, such as restriction of diet and social mobility, may pose risks to the pregnant woman’s health and access to health facilities whereas other remedies such as restricting consumption of meat from animal carcasses and heavy duties, as well as maintaining good social relations, are cultural adaptive mechanisms that indirectly control the transmission of disease and improve maternal health, and thus should not be considered to be exclusively folk or primitive.

**Electronic supplementary material:**

The online version of this article (10.1186/s12884-018-2041-5) contains supplementary material, which is available to authorized users.

## Background

Utilization of professional maternal health services is crucial in improving maternal, foetal and newborn health outcomes. It is estimated that 90% of pregnancy-related maternal deaths can be prevented with timely medical interventions during Antenatal Care (ANC) appointments, emergency obstetric care, safe abortion, delivery and Postnatal Care (PNC) period [1]. As a result, the World Health Organization (WHO) recommends at least four ANC visits, postnatal check-ups within 2 days of birth, and that all deliveries be attended delivered by skilled health providers and that ANC begin as soon as a woman becomes pregnant for essential interventions to be effectively administered [2].

However, maternal deaths remain high in low- and middle-income countries (LMICs) with a maternal mortality rate (MMR) of 239 per 100,000 live births [3]. Untimely, underuse or lack of professional medical interventions have been established as the main contributing factors [4, 5]. Globally, during the period 2007–2014, 64% of pregnant women attended the WHO-recommended minimum of four ANC contacts, with 78% of births being attended by a skilled health professional [6]. Low access to professional maternal care services is worse in LMICs. The Kenya Demographic Health Survey (KDHS) statistics, for instance, indicated that although nearly 90% of women seek ANC in health facilities, 58% of pregnant women made four or more ANC visits, while 20% made their first visit within the first trimester [7]. Many women wait until the last trimester to do this while others make only one visit, thus limiting the benefits that would have been extended to them under the free government maternal care programme [8–10]. Overall, 44% of births in Kenya are attended by skilled birth attendants [11]. The underuse of professional maternal health services is reportedly higher in rural areas [7] and the situation is worse in some parts of the country. For instance, in Uasin Gishu County of Kenya (where this study was conducted), only 22% of pregnant women attended at least four ANC sessions, while only 30% of all births are attended by skilled health staff [12].

In contrast to the limited uptake of professional maternal health services in Kenya and other LMICs, most women make extensive use and reliance on traditional maternal care and remedies, even when they are suffering serious emergency obstetric complications. A study by Kaingu et al. [10] in Machakos County Kenya, for instance, identified a total of 10 pregnancy-related complications and symptoms, including being threatened spontaneous abortion, labour complications and post-partum haemorrhage that are being managed at the mother’s home by Traditional Birth Attendants (TBAs). They further identified 55 plant species that were being used as medicinal herbs for the management and treatment of pregnancy complications. TBAs, mothers-in-law and older female relatives are important community resource persons whom pregnant women routinely consult throughout the course of pregnancy and childbirth, especially in rural areas in LMICs [7, 8, 11]. The situation is made particularly challenging by the fact that most informal maternal care providers, especially in the rural areas, have no formal training in maternal care and child delivery, instead they rely on their traditional knowledge, which is not well known in the literature. There is little evidence that this situation will soon improve because even when mothers seek care at the health facilities, studies indicate that they tend to integrate both western and indigenous knowledge in their understanding of health and medicine [13].

Indigenous knowledge is an integral part of life in rural communities. This knowledge has a great influence on how communities perceive health, illness, causes of disease and consequently their care-seeking behaviours [14, 15]. These traditional healthcare practices could be useful or detrimental. Rather than ignoring and condemning it, this knowledge should be explored, strengthened through research and scientific evidence, documented and disseminated, especially to healthcare providers, so that they can be informed about the actual practices in which women engage during pregnancy and childbirth. To this end, the present study aims to gain insight into traditional antenatal care practices adopted by pregnant women and their implications for maternal health and access to biomedical care interventions. Examining the socio-cultural context of pregnancy and childbirth is important to understanding maternal mortality and health-seeking behaviours.

### Theoretical framework

This study was guided by symbolic interaction and functionalist perspectives. The research findings are analysed through the lens of symbolic interactionism in order to understand how the Kalenjin perceive and respond to adverse pregnancy outcomes. It was established that the Kalenjin women’s responses and behaviours regarding pregnancy complications are directed by the meanings they attribute to these complications. These meanings are constructed and learned through social interactions with other members of society. What is important to realise, however, is that these meanings are based on what is culturally believed to be true and not what is ‘objectively true’ from the perspective of western biomedical models. Hence, these meanings may override and conflict with biomedical realities of health and thus require contextualised interpretations and interventions. Women act in ways that accord with the meaning that they attribute to adverse pregnancy outcomes. Understanding these symbolic meanings, therefore, will assist in comprehending the Kalenjin women’s responses and behaviour towards adverse pregnancy outcomes.

The discussion of the research results was also guided by the functionalist perspectives. These cultural explanatory models of illness, even if based on scientifically incorrect premises, frequently have an internal logic and consistency, which often helps the victim of illness to “make sense” of what has happened, why it has happened and the appropriate disease prevention and treatment therapy to adopt. On the other hand, some of these causal explanations of pregnancy-related illness are deliberate attempts to reduce pregnancy complications but are based on an understanding of disease transmission that differs from the western biomedical models. When the underlying unconscious adaptive significance is effective, these pregnant women will continue adopting the practice even though they may not be aware of the adoptive value of what they are doing, which contributes to the selective retention of these prevention and treatment remedies. For this reason, it is necessary to take into account these unintended, adaptive benefits of various pregnancy practices and belief systems in any attempt to unravel complex patterns of treatment during pregnancy.

However, ethnomedical explanatory models of illness are not always in people’s best interest. Some belief systems and ritual practices appear to have maladaptive effects, which pose medical risks to those practising them. It is therefore important to understand these practices in any given intervention.

## Methods

### Study setting and data collection

The major focus of this qualitative study was on the cultural interpretation of pregnancy complications and the preventive and treatment remedies adopted. This study is part of broader research investigating the socio-cultural context of maternal nutrition and health in rural Uasin Gishu County in western Kenya. Data were collected between April and August 2015 from Kalenjin women, either pregnant or with a child of less than 1 year, seeking care at the government health facilities in the Maternal and Child Health (MCH) care section.

The Kalenjin is the main ethnic population in Uasin Gishu County and comprises eight sub-ethnic groups (the Kipsigis, Nandi, Tugen, Keiyo, Marakwet, Pokot, Sabaot and the Terik) that share a common dialect and similar cultural traits. Among the Kalenjin speakers, each sub-ethnic group has its own distinctive dialect. The Nandi occupies the largest settlement in Uasin Gishu County, followed by the Keiyo.

All 90 public health facilities in the county were included in the sampling frame. Quota and purposive sampling techniques were employed in the selection of a representative sample of health facilities for the study. The selection criteria included ensuring that the health facilities in all the six-quotas (sub-counties) are proportionately represented in the sample. All the health facilities must be in the rural area (outside the municipality territory) and have a catchment population mainly comprising at least 90% Kalenjin patients to enhance cultural homogeneity. This means that areas dominated by other non-Kalenjin ethnic groups and those within the municipal boundaries were eliminated. The last criterion is that the selected facilities should be spatially distributed from each other to diversify responses. In the end, a total of 23 health facilities were sampled for the study.

All the Kalenjin women who come for routine antenatal and post-partum child welfare check-ups in the sampled health facilities were included in the sampling frame. They were recruited at the MCH clinics and in maternity wards. Eligibility criteria for the study participants depended on: being pregnant or having given birth within the last year, a Kalenjin by birth, willing and able to participate in the study, able to give informed consent [16] and willing to be audio recorded. This selection criterion eliminated non-Kalenjin women and those not willing to be audio recorded. Data were collected until the information reached saturation at a sample size of 42 women [17].

Nine key informants, including six TBAs who are also herbalists, one CHW, and one nursing officer in charge of MCH, were also selected for an interview. Quota sampling and purposive sampling techniques were used in the selection of key informants [17]. One TBA from each of the six sub-counties, who was highly mentioned by women respondents who had given birth at home or took herbal remedies during pregnancy, was selected and they could be reached at home or in the market centre. The CHW and nursing officer were selected from one of the largest rural facilities in the county because they are likely to encounter a wide range of pregnancy experiences and challenges given their large catchment area. In total, six TBAs who were also herbalists, one nursing officer offering MCH care, and one CHW were recruited.

#### Data collection

An open-ended interview guide (Additional file 1), divided into four sections, was used to elicit the information from the Kalenjin women. The first section presented demographic characteristics of the respondents including age, educational level, parity, ethnic affiliation and gestational age at the first ANC visit, marital status, and tribal affiliation among others. The other sections contained questions about food restrictions, recommended food, activities restricted and activities encouraged during pregnancy. Every practice mentioned was probed to obtain an insight into the underlying reasons. The respondents were further questioned about their opinions regarding these cultural practices and whether they indeed practised them.

Face-to-face individual interviews were conducted in a private room. Each woman was interviewed once and the interview lasted between 30 and 60 min, depending on her responses. Key informant interviews (KIIs) followed later to provide clarity on the issues raised during the interviews. The KIIs lasted between an hour and 2.5 h. They were also questioned about the kind of advice they give pregnant women and health challenges they face when providing care to pregnant women. Important notes were taken and at the same time responses were audio recorded.

#### Ethical considerations

The study was approved by the National Commission for Science, Technology and Innovation (NACOSTI) in Kenya and a research clearance permit number: NACOSTI/P/15/2335/5353 dated 2 April 2015 was issued to facilitate the research process. As approved by NACOSTI, the permit was then presented to the Uasin-Gishu County Commissioner, County Director of Education and County Director of Health, for their approval to conduct the study in the County. Further, appointments were booked with the respective officers in charge of the various facilities visited. Participation was voluntary. The respondents were informed of the aim of the research, confidentiality and anonymity of their responses, and then gave their signed consent to participate. Permission to audio record the interview sessions was sought from each respondent. Only voices for those who consented were recorded.

#### Analysis

**Fig. 1:**
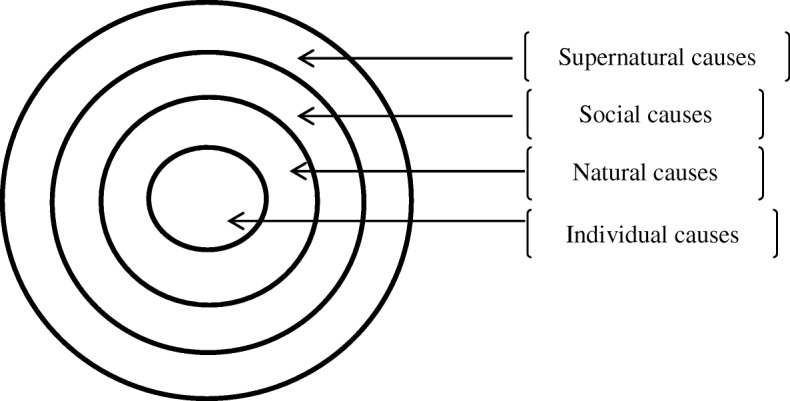
Sites of illness aetiology (Helman, 2000:120)

Recorded responses were transcribed and, together with field notes, were studied by way of content analysis using MAXQDA 12.0.3 software. Helman’s [15] classification of lay-illness aetiologies model was adopted as the initial coding guide. Meanings attributed to various adverse pregnancy outcomes were established in the data and were classified into four major categories based on Helman’s [15] symbolic classification of lay-illness causation aetiologies model: individual, natural, social and supernatural, as illustrated in Fig. 1. The categories were further classified into sub-categories and themes as interpreted below.

##### Individual causes

These include lay theories that locate the meaning of pregnancy complications in the individual woman for “not taking care” of herself in terms of diet, dress, hygiene, lifestyle, relationships, sexual behaviour, smoking and drinking habits, physical exercise, emotions or doing something abnormal or incorrect. An adverse pregnancy outcome is, therefore, evidence of “carelessness” and the woman should feel guilty and responsible for causing it. However, in some rare circumstances, individual causes can result from external forces over which the victim had no control such as bad luck, economic power or hereditary factors.

##### Natural causes

In this category, an adverse pregnancy outcome is thought to be caused by the natural environment, both living and inanimate. Common in this group are climatic conditions, such as excess cold, heat, wind, rain, snow, damp, cyclones, tornadoes, eclipse or severe storms. Others include accidental injuries which originate from the “natural environment”, or are caused by animals, birds, insects, or infections caused by micro-organisms, such as germs, bugs or viruses.

##### Social causes

This category involves blaming other people for causing adverse pregnancy outcomes and is a common feature of non-industrialised and smaller-scale societies, where interpersonal conflicts are frequent. The common forms of these are witchcraft, sorcery and “evil eyes”. In witchcraft, certain people are believed to possess a mystical power to harm others and this power is inherited, either genetically or by membership of a particular kinship group. Sorcery, as defined by Helman [15], is the power to manipulate and alter natural and supernatural events with the proper magical knowledge and performance of rituals, and this is different from witchcraft. Sorcery is often practised among one’s social world of friends, family or neighbours, and is often based on envy. Evil eyes, or a “wounding eye”, relates to the fear of envy in the eyes of the beholder. The possessors of evil eye are usually believed to harm unintentionally and are often unaware of their powers and are unable to control them. The influence of evil eye, as explained by Helman [15], is avoided or counteracted by means of devices calculated to distract its attention, and by practices of sympathetic magic. The social aetiology of illness also includes physical injuries, such as poisoning or battle wounds, inflicted by other people. Furthermore, it can be stress or actions caused by spouse, children, friends, employer or colleagues and neighbours. It can also be contagious diseases transmitted by other people.

##### Supernatural causes

Here a pregnancy complication is ascribed to the direct actions of supernatural entities, such as gods, spirits or ancestral shades. In the gods aetiology, illness is described as a reminder from God for a behavioural lapse or sinful behaviour. The cure in this case involves acknowledging the sins and vowing to improve one’s behaviour. In the case of spiritual causes, disease-bearing spirits strike unexpectedly causing a variety of symptoms in their victims. Their invasion is unrelated to the individual’s behaviour, who is therefore considered blameless and worthy of sympathetic help from others. In the case of ancestral shades causes, a pregnancy complication is ascribed to spirits of the ancestors whom they have offended and diagnosis takes place in a divinatory séance.

## Results

### Socio-demographic characteristics of the respondents

A total of 42 female respondents were interviewed, of whom 14 were pregnant and 28 were post-natal (Table 1). Half of the respondents had acquired primary education. All respondents were age 18 years and above and more than 26 respondents were between 20 and 29 years of age. Many respondents (*n* = 30) were married and almost all worked in the informal economy as subsistence farmers, homemakers or small entrepreneurs. Only 13 of the respondents were first-time mothers and the other 29 had 2–8 children. For the recent pregnancy, three respondents did not seek any ANC from the health facility. For those who attended ANC at the health facility, only three did so in the first trimester while the majority started after 6 months of pregnancy.

**Table 1:** Demographic characteristics of the respondents

Characteristic	Categories	Number of cases (*N* = 42)
Maternal status	Ante-natal	14
	Post-natal	28
Parity	First Pregnancy/child	13
	1–3	15
	4–6	11
	7–8	3
Gestational age at the first ANC visit	Never visited	3
	2 months	3
	4 months	3
	5 months	6
	6 months	16
	≥ 7 months	11
Age of respondents (years)	≤ 19	4
	20–24	16
	25–29	10
	30–34	7
	≥ 35	5
Marital status	Never married	8
	Currently married	30
	Separated	2
	Widowed	2
Educational level	Primary Education	21
	Secondary Education	16
	Tertiary Education	5
Occupation	Student/pupil	4
	Business	7
	Farming	26
	Formal employment	1
	Other	4
Sub-ethnic groups	Nandi	31
	Keiyo	4
	Marakwet	5
	Terik	1
	Kipsigis	1

### Perceptions of and responses to adverse pregnancy outcomes

Several perceived causes of pregnancy complications and the remedies adopted were identified and classified into four major categories based on Helman’s [15] classification of lay-illness aetiologies model as illustrated in Fig. 1. Further, sub-categories and themes that emerged were established and are presented in Table 2.

**Table 2:** Interpretation of pregnancy complications and remedies

Category (Cause)	Subcategory (Sub-cause)	Theme (perceived causes)	*N* = 42 (%)	Theme (perceived remedies)
Individual causes	Physical activities	Doing heavy duties	25(60%)	Avoid doing heavy duties
		Having sexual intercourse during pregnancy	10(24%)	Abstain from sexual intercourse during pregnancy
		Idling	9(21%)	Do light duties
		Standing or peeping at the door/window way	6(14%)	Either walk out quickly or stay
		Running or walking long distances	5(12%)	Avoid running and long walks
		Oversleeping	4(10%)	Avoid over sleeping
		Boarding a motorbike	2(5%)	Avoid using motor bikes
		Sitting and sleeping style	2(5%)	Sit with legs straight, Sleep on the side, not on back
		Dress code	2(5%)	Avoid dressing accessories around the body including necklace, belts or clothes with bands around the waist
	Diet	Eating restricted food (eggs, avocado, meat, oily food, fresh milk, cold ugali cold water, alcohol and cigarettes)	41(98%)	Avoid restricted food
		Not eating recommended food and herbal medicine (traditional vegetables, milk, liver, ugali)	41(98%)	Eat recommended food
				Use herbal medicine
Natural Causes	Sickness and hereditary complications	Severe cramps during menses	8(19%)	Seek herbal treatment
		Wrong foetal presentation	3(7%)	Seek TBA care to turn the foetus
		Malaria	2(5%)	Take preventive herbal remedies
				Only take prescribed drugs
		Amount of hair and sex of the foetus	2(5%)	Seek TBA advice/treatment
		Yellow fever	1(2%)	Seek herbal treatment
Social world	Getting in contact with dangerous people	Contact with a woman who had an abortion, or whose child died recently, or who had a wrong foetal presentation during birth, multiple births or who develops severe cramps during menses, or who have evil eyes	10(24%)	Reduce movements
				Avoid going to public places
				Use protective herbs
	Social relations	Quarrelling with/abusing someone	5(12%)	Maintain peaceful relations
				Confession and repentance
		Made to cry or stressed	2(5%)	Avoid emotional events
Super natural causes	Evil spirits	Eating meat of a “misfortune animal”	16(38%)	Avoid eating “evil meat”
		Killing an animal	12(29%)	Avoid killing any animal
		An animal crossing your way or meeting two snakes on your way	3(7%)	End the journey whenever one meets world animal, mostly a snake, on the way
	Gods	Laughing at a deformed person	8(19%)	Do not lough at a deformed person
	Ancestral spirits	Viewing or burying a dead person	11 (26%)	Avoid burials
				Do not view a dead body
		A relative committed evil actions towards the ancestor	4 (10%)	Conduct a reconciliation ritual

### Individual causes

Individual cause is one of the factors that strongly emerged in this study. Under this category, pregnancy complications are seen as evidence of the pregnant woman’s carelessness and as a result she should feel guilty about her incorrect practices during her pregnancy. These are particularly related to physical activities and diet.

#### Physical activities

One of the restricted activities that strongly emerged in the study, as reported by 60% of the respondents, is that a pregnant woman should be exempted from performing “heavy duties”. Heavy duties that were commonly forbidden according to the respondents refer to activities that involve bending for long hours, carrying heavy loads, fetching water from an open well, “scooping soil” (digging), carrying soil and smearing mud houses. Other activities reported include: hand washing many clothes, splitting firewood, collecting water from the river using a heavy container (e.g. 20 kg), or carrying a heavy load of firewood. Heavy duties are believed to cause lower back pain that may cause a miscarriage, pre-term birth and excessive bleeding after birth or low birth weight. Splitting firewood is also believed to make the baby’s fontanelle abnormally large, which is dangerous because it is believed to cause its death.

*Heavy duties like digging, splitting firewood should be avoided because it drains all the energy from the lower back making it ache and become weak. This is dangerous….it can make the baby come out* [abortion].

To avoid lower back pains that cause a miscarriage, it is perceived that a pregnant woman should not only avoid heavy activities, but also seek herbal treatment. The herbs are believed to strengthen the lower back and thus minimise back pains during pregnancy and after the birth.

*When a woman is pregnant, she should not overwork herself with housework or digging. This is why some of them come to me complaining of back pains. I give them pain relief herbs and I normally tell them to completely avoid overworking themselves.* **(**Herbalist 2)

Some respondents felt that fetching water from an open well is risky; a woman might lose her balance and fall into the well. Collecting water from the well is discouraged in the first 4 months of pregnancy, because at this stage, pregnancy is still considered immature hence fragile and easy to abort. Thus, such activity can be undertaken after a pregnancy is 5 months or more. A pregnant woman may also carry a light load (10 kg or less) that she can easily lift without being assisted.

*Sometimes there are women when giving birth, legs of the baby come out first instead of the head or those who experience severe cramps during menses. Such a woman should not assist you in lifting the load to your head or back when you are pregnant. You will also give birth like her or have an abortion.*

A pregnant woman is also advised to abstain from having sexual intercourse, as reported by 24% of the respondents. However, the period of abstinence varied; to some, abstinence should begin the moment you realise you are pregnant, to others from 6 months onwards. The semen is believed to make the baby dirty, because it sticks on the baby’s skin and scalp, creating a white substance. Thus, the baby must be washed with herbs for several days to clear it off. The “semen” is also believed to be dangerous for the foetus because it is thought to block the nasal passages and this could make breathing difficult and subsequently cause death. It is also thought that the “semen” can be swallowed by the foetus and remain stuck in the chest, causing a wheezing chest. The condition is believed to be more dangerous if the baby is a boy because males are considered less active in the uterus and cannot wash it off into the amniotic fluid. A woman who did not abstain from sex will be detected during birth because “she will be messy with semen” and nobody will be willing to assist her delivery, including the midwife. Instead, they will use plastic bags as gloves in assisting delivery, and the woman and her husband will be abused and ostracised.

*…..ejaculations of the man are dangerous to the baby and messy, even those facilitating your birth might run away and leave you, they do not like looking at those things. They will be forced to use plastic bags to remove the baby and give you to wash it by yourself. They only assist you well if you give birth to a clean baby.*

Some respondents, including one of the CHWs, reported that they use condoms during pregnancy to prevent the semen from reaching the baby.

On the other hand, a pregnant woman should not be idle (21%) and should not oversleep (10%) but perform light duties. Light duties include washing a few clothes, grazing cows, weeding a small plot, or taking a nature walk. It is believed that, when a woman is idle or asleep, the foetus also becomes idle, inactive and docile because it tends to sleep a lot. A docile foetus, according to the respondents, is not strong enough to assist the mother when she is pushing during delivery, resulting in prolonged labour. Similarly, if a pregnant woman is idling or sleeping a lot makes her muscles will be weak during labour and she will not find it easy to push out the baby.

*I was told not to sleep too much but to be active in doing light duties like grazing animals and digging but not too much of it. It makes the baby play well and be positioned in the right part of the womb; otherwise I will have trouble during birth. The pain will extend for long.*

*Domestic duties like washing utensils, and when your body is okay you can go to the farm. It helps blood to flow very well, and the body to function well, but if you just stay idle it can even bring you diseases like high blood pressure and diabetes.*

A pregnant woman, as reported by 14% of the respondents, should not peep through or stand in the doorway or window of a house. If she has a tendency to do this and return, during labour, the baby will also peep though the cervix, trying to come out, but instead return back into the uterus, thus prolonging labour. To avoid the chances of prolonged labour, a pregnant woman should decisively either stay inside or just walk out of the house and avoid standing in the doorway.

A pregnant woman should also avoid running or walking long distances, as reported by 12% of the respondents:

*One should not run when pregnant, she can accidentally fall down causing the pregnancy to pain* [contractions] *before term.*

Other restricted activities during pregnancy that were reported by less than 10% of the respondents include boarding a motorbike (the means of transport commonly used in rural areas), the dress code, sitting style and sleeping positions. High heels are considered dangerous because they might cause a woman to fall, and that could lead to pre-term contractions/miscarriage. Pregnant women should also avoid clothes with a band around the waist, such as skirts, trousers and belts. Bands around the waist are believed to make the umbilical cord twist around the baby during birth, which they believe may cause stillbirth. A pregnant woman should sit with her legs straight and apart. Sitting with her legs twisted or bent, they believe, will block the birth canal and result in obstructed labour.

#### Diet

Pregnancy complications were also thought to be caused by the wrong diet during pregnancy. Some foods, as reported by 98% of the respondents, were considered to jeopardise a pregnancy if consumed in excess, while others can endanger pregnancy if they are not eaten in sufficient quantity. Thus, a pregnant woman is required to eat sparingly and selectively. High-protein and energy-rich foods were believed to make the foetus grow big. A big foetus cannot be pushed out easily and will cause an obstruction, thus prolonging labour and possibly lead to caesarean section (CS). CS is believed to be a risky process in the sense that it halves the chances of mother or child survival, unlike an uncomplicated vaginal delivery. The big foetus also results in a prolonged labour and may cause tears by forcing itself out. Eggs, avocado, and oily food were commonly reported as needing to be avoided or restricted.

*I was told not to eat strong food like eggs and avocado. The baby will grow big and later will give me problems when giving birth and I can even be operated.*

Other foods of this kind that should be eaten sparingly include: meat, fresh milk, cooked bananas, and cold *ugali*. TBAs/herbalists prescribe herbs to regularise the size of the baby especially if it is judged to be too big.

Consumption of alcohol and cigarettes is restricted during pregnancy because it is believed to result in low birthweight and mentally retarded babies. Furthermore, when in labour, a pregnant woman should not drink cold water. It is believed to freeze contractions and thus lead to prolonged labour.

Some foods were believed to be good for pregnancy, hence encouraged to be eaten in plenty. According to most respondents, a pregnant woman is recommended to increase her blood volume and energy. A woman with less blood volume is considered not to have strength to push out the baby, will bleed to death during labour, and might require blood transfusion, which is believed to be risky. Therefore, lots of the foods that are believed to increase the amount of blood should be eaten for the entire period of pregnancy. These include indigenous vegetables (mostly leafy greens, including pigweed, black nightshade, spider plant, spinach, white vine spinach, pumpkin leaves and cowpea leaves), fruits, liver, animal blood, milk (especially when mixed with animal blood), red beans and their soup, meat, porridge made of finger millet flour, red soil or red stones and some traditional herbs.

*When your blood is less, it will only help you to get a baby, thereafter; you will bleed until your body dries up and you die.*

A pregnant woman is also required to be stronger during pregnancy. It is believed that a weak woman will not be able to push the baby out.

*If you do not feed well on the pregnancy recommended food, you will not have strength for pushing out the baby during birth. You will be weak, you can even faint. They will demand an operation in order to remove the baby. With the operation you might die.*

It is therefore recommended for her to eat “strong” energy-giving food during the pregnancy and minimise “less” energy food. *Ugali* and porridge were the commonly reported foods believed to be rich in energy. Others were milk, rice, sweet potatoes, Irish potatoes and herbs. However, rice and Irish potatoes are regarded as ‘less energy-giving food’ that should be eaten only once in a while.

Use of preventive herbs during pregnancy was reported by the majority of the respondents, mainly to prevent mother-to-child transmission. A TBA presented 10 different herbal medicines that are boiled together for pregnant women to drink, whether sick or not. Hence, a pregnant woman will be held responsible for any miscarriage if she refuses to take these preventive herbs.

### Natural causes

Some aspects of the natural environment were also believed to cause pregnancy complications, though not reported by as many respondents as other aspects of Helman’s model. The aspects of natural causes that emerged in this study were mainly infectious and hereditary diseases. Severe pre-pregnancy menstrual cramps (*chepsaliat*), as reported by 19%, are believed to cause miscarriage or pre-term contractions during the first months of pregnancy. Therefore, it is thought that girls who experience severe pre-pregnancy menstrual cramps are likely to abort within 4 months of conception if they do not seek herbal treatment before conceiving.

*I had a miscarriage at two and a half months in my previous pregnancy, my grandmother told me it was caused by cramps. When I was a girl, I used to have severe menstrual cramps.*

Severe menstrual cramps, as explained by the herbalist, are inherited and are extended to pregnancy and are believed to pierce the capillaries supplying blood to the uterus resulting in “blood leakage” that supposedly causes spontaneous abortion mostly within the fourth month of pregnancy.

To avoid the chances of early miscarriage, girls who experience pre-pregnancy *chepsaliat* are highly recommended to seek herbal treatment before conception. However, if the woman notices unusual contractions or spots of blood and seeks immediate herbal attention, “the capillaries can be sealed” (spontaneous abortion can be controlled) as explained by a TBA/herbalist:

*….If someone ignores the herbs and instead opts for hospital treatment before taking the herbs, she cannot control the bleeding even if I give her the herbs. That one is beyond repair, the herbs cannot help her. Whoever ignores these herbs aborts.* (Herbalist 2)

If a pregnant woman falls sick, her illness is also believed to be transferable to the foetus causing foetal death (miscarriage or perinatal death). The reported diseases were malaria and yellow fever. As a result, maternal sickness or the possibility of mother-to-child transmission should be prevented by periodically drinking herbs or be treated using mild herbal medicine. Hospital medicines are acceptable but believed to be ‘too strong’ and may cause spontaneous abortion, hence herbs were preferred.

*When I miscarried my previous pregnancy, the TBA told me a different causal reason from my grand mum…. The cause she told me was yellow fever. When I conceived this second pregnancy, she gave me preventive herbs. I started taking from the time my pregnancy was 1 month old. I used to take them daily for 5 days per month until I finished 3 months then she terminated the dose.*

Other natural causes were associated with the foetus itself. For instance, the sex of the foetus and its amount of hair were considered a determining factor of the mother’s feelings and physical status during pregnancy. Female foetuses are believed to give the mother nausea, lack of appetite, feeling sickly and make her thin and moody, whereas male foetuses are believed to make the mother crave certain types of food, increased appetite, look healthy, strong and jovial. Abundant foetal hair is associated with heartburn to the mother. It is believed that the long hair stretches to the throat causing the irritation.

Numbness of one leg, which complicates movement during pregnancy, is thought to be caused by the abnormal presentation of the foetus and this is believed to cause complicated delivery. TBAs are believed to have inherited skills to massage the uterus and correctly reposition the foetus, hence relieving discomfort to the mother and making birth easier.

### Social causes

The social-based explanations that emerged strongly from the study, as reported by 24% of the respondents, is that a pregnant woman should not contact “dangerous people”.

Pregnancy complications were believed to be contagious. For instance, a woman who had ever had an abortion or whose child died recently is considered to be contagious, and if she is in contact with a pregnant woman or her shadow falls on the body of a pregnant woman, it is believed to cause miscarriage, stillbirth, and perinatal death or make the woman sick. Other women believed to be dangerous and who should be avoided included those who had a breech or traverse presentation during birth or a multiple birth, or who experience severe menstrual cramps. This can cause pre-term contractions that might cause spontaneous abortion.

*I was told not to meet someone whose child died or to get near her or the one who encountered an abortion. If she gets close to you, your baby might also come out* [abortion] *or you can even fall sick.*

Some people are known to have evil eyes that can cause a miscarriage if they meet a pregnant woman. Therefore, a pregnant woman should stay away from such people. Some of these “dangerous” people are known, while others are not. Hence, the main way to reduce the chances of meeting them is to restrict movement outside the homestead, avoid walking along public roads, and going to crowded places such as market centres or other social gatherings.

*I was just told when someone is pregnant like me now; I should avoid going where there are a lot of people.*

*Women are not allowed to visit to places when pregnant. They can meet evil people that can cause problems to the foetus.* (Herbalist 1)

As a remedy, herbs are available in a roasted powder form, called *bosarok*, which is to be licked every morning. Pig oil can also be applied on the pregnant woman’s face and tummy, especially when she is going on a journey, to counter evil eyes. Such herbs were commonly reported by respondents during the study.

*When you wake up in the morning, the first thing you should do even before you wash your face is to lick the ‘bosarok’* [herbs]. *The timings and location of these women is not known. She might come to your house early in the morning to borrow tea leaves for breakfast. These herbs keep the baby safe in the stomach [womb]. If you use them as reserved, these spirits cannot attack the baby.*

### Social relations

A pregnant woman should not exchange utterances or abusive words with anyone. This is because the opponent might “abuse her badly”. The feared abuse mostly reported was “you will give birth but you will not hold that baby”, referring to a stillbirth. If a pregnant woman is abused with these words, it is believed that this woman will have a stillbirth, or her baby will die shortly after birth. Moreover, the husband of a pregnant woman or any other close person should not do anything or utter words that might annoy or make her cry or emotionally stressed.

*When pregnant, you should not quarrel or exchange words with anybody even if you find a person red handed backbiting you or doing something wrong to you. You simply stay silent and assume that nothing is wrong. If you start exchanging words, the person might abuse you badly and at the end you will give birth and your baby dies.*

*It is not good for a pregnant woman to cry. This is like moaning for her death or that of her unborn child. She might die or give birth to a dead baby.*

Therefore, to avoid complications that may lead to a stillbirth or perinatal death, it is recommended to maintain peaceful relationships by avoiding quarrels. Similarly, a pregnant woman should not be abused or offended even if she transgresses anyone. Should she be abused or offended accidentally, the offending person should apologise immediately. If a pregnant woman had a dispute with someone, and this was not resolved, and she develops a complicated labour, the person concerned or who caused her stress will be sought to come and confess, in order to ease labour and facilitate birth. The offender will be held accountable for any calamity and this will cause the person emotions of shame and guilt. Therefore, a pregnant woman is expected to be respected by members of the community.

#### Supernatural causes

In this category, explanations of pregnancy complications were ascribed to supernatural causes, mostly spirits and to some extent ancestral spirits and gods.

### Evil spirit of the dead

A pregnant woman is not supposed to eat meat of an animal carcass, as reported by 38% of the respondents. Such meat was believed to possess evil spirits that are transferable when eaten. The meat commonly reported to be avoided is an animal that was slaughtered because it suffered and died from pregnancy-related complications, such as placental retention, haemorrhage, an abortion or stillbirth. If eaten, it is believed that the “bad blood” that caused such complications is transferred, causing very similar complications to a pregnant woman who consumed it. Similarly, an animal that was slaughtered because it was sick, for unknown reasons, struck by lightning or by strangling itself with its umbilical cord, should not be consumed. The former is believed to cause maternal death whereas the latter will make the umbilical cord coil around the baby’s neck resulting in stillbirth. Thus, a pregnant woman should be aware of the meat she eats. However, if she eats it without knowing its provenance, cleansing rituals must be performed to clear off the spirits of the bad blood.

*In case there was a goat which died with kids in the stomach, that meat should not be eaten. Or a cow that died during labour, for example the calf came out but placenta retained she should not eat its meat. If she eats it, the placenta will also be retained during birth or she will die just like that animal.*

On the other hand, as reported by 29% of the respondents, a pregnant woman or her husband should not kill any animal (wild nor domestic) or insects. Instead, they should get someone from the neighbourhood to do it for them. Otherwise it is believed that she will give birth to a baby with similar features of that animal. Unless they conduct rituals that involve giving the baby the name of that animal, the baby will retain those features to maturity, some of which are dangerous. The commonly mentioned animals were snakes, cats, dogs and chicken, and the killing was believed to cause disabilities such as blindness, lameness or even death.

*I was told not to kill a snake; my baby will look like a snake… will have red eyes that look away from each other and unstable neck, just like a snake. They will call the baby ‘Kiberen’ continuously until the features disappear. The baby will also cry like a cat if you killed a cat or walk using toes like dog with a funny head if you killed a dog.* (TBA 3)

Similarly, 26% of the respondents further reported that a pregnant woman is not allowed to view a dead body. The commonly reported forbidden act is viewing the body while inside the coffin or inside the grave. Traditionally, they were not allowed to even attend the funeral as reported by a key informant. If a person dies in the homestead, she moves away until the burial rituals are completed. The husband of a pregnant woman is not allowed to take part in digging the grave and burying of the dead. Many respondents could not understand why it is prohibited, yet they obeyed the custom. However, some respondents reported that a funeral is emotional and it worsens when viewing the body or burying the corpse. These emotions can make one faint and collapse which can cause damage to the uterus resulting in miscarriage e, stillbirth or maternal death.

### Gods

Foetal deformity, as reported by 19% of the respondents, could arise if the pregnant woman makes fun of a person with a physical or mental disability. It is believed that this could result in God afflicting the infant with a similar disability.

Other beliefs ascribed to supernatural causes that emerged in the study were based on ancestral spirits, especially if a close relative of the pregnant woman’s husband committed an evil act to a deceased member of the family and did not reconcile before he/she died. In this case, therefore, when a pregnant woman undergoes prolonged labour, male elders will be called to conduct reconciliation rituals at the graveyard of the deceased.

## Discussion

This study examined the lay meanings ascribed to adverse pregnancy outcomes and the adopted treatment and remedies among the Kalenjin in rural Uasin-Gishu County in Kenya. A healthy pregnancy is perceived as a process that pregnant women are able to manipulate by observing behaviours, and taking remedies against natural, social and supernatural forces during pregnancy. The explanatory models of these local meanings are deliberate attempts to reduce and prevent pregnancy complications but are in many cases based on an understanding of disease transmission that differs from biomedical explanations. Some remedies are beneficial, others make no sense at all, while others to some extent can even be detrimental to maternal health as indicated below.

### Restricting geographical mobility

In this study, pregnancy complications are attributed to evil people, so pregnant women are confined to the homestead to avoid contracting evil people and are encouraged to apply medicinal herbs to counter evil. Restrictions on geographical mobility reduce vulnerability to contracting infectious diseases. Pregnant women are at a higher risk and more susceptible to or more severely affected by infectious diseases because of the unique “immunological” condition caused by pregnancy. Infections contracted during pregnancy are in most cases associated with maternal death, stillbirth, spontaneous abortion and pre-term birth, hence this is a good practice that should be enhanced. However, the fears of meeting evil people can result in disruptive or non-use of public health facilities by avoiding going outside. Beliefs in witchcraft, poisoning or spiritual attacks are a major component of African cultures’ explanatory model of pregnancy complications and illness and have been established as a major barrier to accessing health facilities for care [18, 19].

### Use of herbal medicine

Preventive herbs are believed to protect pregnant woman from getting sick and from transmitting sickness to the foetus. Naturally, pregnancy is a state of immunological weakness, and therefore of increased susceptibility to infectious diseases. Improving the immune system is one of the most important ways of protecting the mother against the environmental infections and preventing damage to the foetus. Malaria In Pregnancy (MIP) is a major public health problem in areas of sub-Saharan Africa, including parts of Kenya, where malaria is endemic, and has important consequences for the birth outcome. Similarly, the risk of pre-term delivery and of miscarriage was found to be high in mothers with HIV and with MIP occurring within 2 weeks of delivery [20]. Use of minerals, plants, and animal products as medicines during pregnancy is a common practice in Kenya, especially among the Kalenjin, to treat pregnancy-related complications and symptoms [10, 21, 22]. Some herbal plants have been confirmed to have antibacterial, anti-inflammatory, antimicrobial and antimalarial properties [23]. However, the safety and efficacy of many of these medicinal herbs, especially during pregnancy, when the human body is vulnerable, still needs to be established.

### Avoid sexual intercourse during pregnancy

Ante-natal sexual taboos, which prohibit couples from having sexual intercourse from the second trimester of pregnancy, were also established in the study. In African societies, abstinence from sexual intercourse during pregnancy and for some period after childbirth is a common phenomenon, and is believed to be associated with contamination that might be harmful to the unborn baby or the husband and that the mother is considered to be too fragile to have sex [19, 24, 25]. These women may not justify sexual abstinence during pregnancy in biomedical terms, but scientifically the latent function of this custom is to prevent the transmission of sexually transmitted infections (STIs) to a pregnant woman. STIs, including HIV/AIDS, among pregnant women are common in Kenya [26–28] and have been associated with a number of adverse pregnancy outcomes, such as spontaneous abortion, ectopic pregnancy, pre-term delivery, low birthweight, stillbirth, postpartum sepsis, and congenital infection [28], thus justifying the custom. However, encouraging sexual abstinence during pregnancy might result in men seeking extra-marital sex and might thereby increase the spread of STIs. Some pregnant women in this study opt to use condoms during sexual intercourse to overcome the complications, a practice that needs to be encouraged.

### Minimise emotional stress

The belief that a pregnant woman is not supposed to experience emotional stress was also established in this study. Morris [24], in his study in south-eastern Madagascar, also established that prolonged labour and other delivery complications were viewed as resulting from an unsettled feud with someone, especially one’s parents or partner, and this can be prevented by resolving feuds or obtaining benediction before or during delivery in order to prevent or resolve complications. A belief that quarrelling, fighting and emotional stress during pregnancy could lead to adverse pregnancy outcomes was also established in some communities in Ghana and Zambia [24, 27]. This is an adoptive practice that is supported by scientific explanations. It not only enhances social cohesion and harmony, but is also beneficial to the wellbeing of the mother and unborn baby. Studies have confirmed that gastritis, hypertensive disorders, pre-term labour, prematurity birth, low birthweight and perinatal death are significantly more frequent among emotionally stressed women [29, 30].

### Avoid heavy duties and risky activities but do not stay idle

A number of activities were considered taboo during pregnancy because of their potentially harmful effect on pregnancy and the foetus. Heavy duties such as collecting water from an open well, splitting firewood, digging and bending for long hours as well as running and standing at the doorway are highly discouraged during pregnancy. Some of these activities endanger the pregnancy and others can cause physical injuries, in the case of a fall. Activity during pregnancy is recommended, but heavy duties should be avoided, and enough rest periods assured, especially in late pregnancy and among women with a high-risk profile [31]. Strenuous work, especially involving long hours of standing and walking, seem to have a negative influence on the growth of the foetus and increase the risk of pre-term delivery [31].

On the other hand, physical activities during pregnancy are encouraged. A pregnant woman is recommended to stay active by engaging in light duties. Bed rest during pregnancy decreases the risk of developing severe hypertension [32] and can improve foetal growth [32]. A belief in maintaining light duties during pregnancy was also established in Zambia [33] and Ghana [34]. Hence this belief and practice of avoiding heavy duties and engaging in light physical activities to exercise the body should be encouraged with scientific explanations.

### Observing an appropriate diet

Pregnancy food precautions as a concern for a healthy pregnancy and birth outcome were established in this study. Pregnant women are restricted from consuming protein-rich food such as eggs, meat, and fresh milk for fear of CS arising from big babies. More than half of the respondents in this study reported that they were small-scale farmers and the reported farm produce were vegetables, milk and chicken. This means that eggs, milk and meat are the major sources of protein that are readily available and accessible in their environment are restricted. Protein deficiency is often associated with spontaneous abortion [35] hence there is need for health practitioners to raise awareness against this belief for the benefit of the pregnant women and their children. However, in case of cultural sensitivity against this practice, culturally acceptable high-protein foods should be encouraged. Pregnancy food taboos as a way of restricting the foetus from growing too big in order to facilitate an easy birth as found in this study was also established among other communities in Kenya, Ghana, Ethiopia and south-east Nigeria [36–41].

Consumption of the meat of an animal carcass is a taboo that is highly condemned as reported by 38% of the respondents. This is a good practice that needs to be encouraged because studies have confirmed that bacteria in animal carcasses can lead to human illness if consumed [42].

On the other hand, pregnant women were encouraged to eat certain foods that were believed to be important in increasing blood volume and facilitating easy labour and birth. Food believed to increase blood volume include traditional green leafy vegetables, liver, animal blood, fruits, milk, beans, and fish. Iron deficiency anaemia during pregnancy has long been implicated in miscarriage [43] and is the major contributor to post-partum haemorrhage (PPH), which is the single leading cause of maternal mortality and morbidity in low-income countries [44]. More than half of all maternal deaths occur within 24 h of delivery, mostly from excessive bleeding, thus, women’s belief is justified and the foods believed to increase the “volume” of blood are truly rich in iron, hence the practice needs to be encouraged. A belief in the importance of iron-rich food in a successful pregnancy outcome was also established in Ghana [45] and Kenya [37, 38].

Pregnant women were also encouraged to consume foods believed to make a pregnant woman strong, because energy is associated with easy birth. Food believed to make a woman strong include *ugali* and porridge made from finger millet mixed with sorghum, and traditional vegetables, milk, traditional herbs and meat. Some of these foods are indeed energy-rich and studies have confirmed that the risk of spontaneous pre-term birth and low birthweight increases in women with limited weight gain [46], hence this practice needs to be encouraged. The importance of acquiring strength during pregnancy was also established in Ghana [45].

### Remedies against supernatural attacks

In this study, pregnant women are restricted from killing any animal. The commonly reported animals that should not be killed because of supernatural reasons include dogs, cats, snakes, chicken and insects. These animals if agitated may produce chemical toxins through a bite or sting, which can kill as a defence against the attacker; hence it is a good practice to avoid them. According to Huntingford [47], among the Nandi, it is believed that the spirits of dead ancestors or relatives return to this world to visit their people and they normally travel in the bodies of snakes, moles, or rats, which they use as vehicles to carry them to and from the houses of the living. If a snake or these animals go to people’s houses especially at night or to the pregnant woman’s bed or house, they may not be killed, as it is believed that they personify the spirit of a deceased ancestor or relative, and that have been sent to indicate to the woman that her next child will be born safely. Instead milk is poured on the ground for it to drink, and then it is allowed to leave the house. If the animal is killed, it will be unable to get back to spirit-land without great difficulty and will be very angry with the offender, resulting in punishment from the spirit. Usually this punishment takes the form of death or illness in man or beast.

In the event that such animals are killed, a pregnant woman is expected to undergo cleansing rituals to end any calamity that is likely to follow her after exposure. The belief that killing of animals will lead into birth of visually impaired child was also established by Ogechi and Ruto among the Gusii and Nandi of Kenya and these were corrected by conducting a ritual [48]. Scientifically, it has been established that maintaining health through spiritual harmony and spiritual healing reduces anxiety, tension and stress associated with (or causing) illness if the patient has faith in the healer, thus increasing the chance for the prescribed therapy to be effective [49]. However, these spiritual healings may coexist or compete with biomedical treatment, thus negatively affecting access and use of health facilities.

### Limitations and strengths of the study

This study focused only on pregnancy-related beliefs up to the point of birth. Post-pregnancy beliefs were beyond the scope of this study and hence need follow-up research. Further exploration of views is necessary to explain some of the Kalenjin perceptions and practices. We are also aware that the respondents who were interviewed were those who had sought care at health facilities. They might have different views from those who do not seek such care. Similarly, respondents were selected from rural Uasin Gishu County health facilities, and we do not know the extent to which respondents attending urban facilities might have different perceptions. However, this being an exploratory qualitative study provides a deeper understanding of the phenomenon on question.

## Conclusion

This study aimed to gain insight into the socio-cultural perceptions of maternal morbidity, mortality and other complications associated with pregnancy and childbirth and to establish how these perceptions influence maternal health and care-seeking behaviours. This study has shown that traditional beliefs of health and illness continue to shape reproductive and maternal health practices among the Kalenjin women of Kenya. This is shown by the fact that many respondents attribute maternal health complications to taboos of pregnancy and childbirth, such as acceptable foods to eat, places women can and cannot go, activities in which women should or should not engage during pregnancy and the spirits of dead animals and ancestors. Participants also reported using traditional herbal medicine as a healing and preventive remedy during pregnancy and after birth. These cultural taboos and beliefs allow Kalenjin women to make sense of their maternal experiences, and to care for their health, hence shaping their care-seeking behaviours. Some of the cultural remedies adopted to prevent morbidity and mortality during pregnancy are cultural adaptive mechanisms that indirectly control the transmission of disease and improve maternal health and thus should not be considered to be exclusively folk or primitive. Such activities include: condemning the consumption of meat from a dead animal, avoiding heavy duties and going to crowded areas, maintaining good social relations and avoiding emotional events such as funerals. However, other cultural remedies such as restricting diet and geographical mobility, may pose risks to the pregnant woman’s health and access to health facilities. The delay in deciding to seek maternal care is a result of women’s failure to recognise symptoms and maternal health problems as potentially needing hospital care, and this failure stems from culturally informed perceptions of symptoms of maternal morbidity and pregnancy complications that differ significantly from biomedical implications.

## Additional file

Additional file 1: Interview guide for pregnant and postnatal women seeking maternal care at a health facility. (DOCX 39 kb)

## Abbreviations

ANC: Antenatal care
ASALI: A sustainable approach to livelihood improvement
CHWs: Community health workers
CIS: Centre for International Studies
CS: Caesarean section
FGM: Female genital mutilation
KII: Key informant interviews
MCH: Maternal and child healthcare
MIP: Malaria in pregnancy
MMR: Maternal mortality rate
NACOSTI: National Commission for Science, Technology and Innovation
PNC: Post natal care
SEKU: South Eastern Kenya University
STD: Sexually transmitted diseases
STI: Sexually transmitted infection
TB: Tuberculosis
TBAs: Traditional birth attendants
VU: Vrije Universiteit Amsterdam
