# Supplementary material for: Perceived causes of adverse pregnancy outcomes and remedies adopted by Kalenjin women in rural Kenya
Source: BMC Pregnancy Childbirth. 2018 Oct 19;18:408. doi: 10.1186/s12884-018-2041-5 (PMC6194609; doi:10.1186/s12884-018-2041-5)
Supplement: Supplementary file 1 — Interview guide for pregnant and postnatal women seeking maternal care at a health facility. (DOCX 39 kb) [file 12884_2018_2041_MOESM1_ESM.docx]

| **INTERVIEW GUIDE FOR PREGNANT AND POSTNATAL WOMEN SEEKING MATERNAL CARE AT A HEALTH FACILITY**  DATE OF INTERVIEW--------------NAME OF FACILITY----------------------------TYPE OF FACILITY ---------------------  SUB-COUNTY--------------TIME STARTED---------TIME ENDED----------ESTIMATED LENGTH OF INTERVIEW----------  INTERVIEWER’S NAME----------------------------------SIGNATURE------------------------------------  **PART A: IDENTIFICATION AND INFORMED CONSENT**  **Ice Breaker Question**  Hi, I can see you have been blessed with a pregnancy, tell me how are you faring on with it?  ***Informed Consent Statement for Survey Respondents on Food beliefs and Practices***  Hello my name is ----------------------------------we are a team from VU University Amsterdam in the Netherlands doing a PhD research study to understand the food beliefs associated with pregnancy. We would very much appreciate your participation in this survey. The information you provide will assist designing intervention programs aimed at enhancing appropriate maternal nutrition intake. You are encouraged to participate freely in this discussion. Any information you provide will be kept confidential and will not be shown to anyone other than members of our survey team. Participation in this survey is voluntary, and if we should come across any question you don’t want to answer, just let me know and I will go on to the next question; or you can stop the interview at any time. However, we hope that you will participate in this survey since your views are important. The interview usually takes 30-60 minutes to complete. If you have any question regarding this research, please feel free to ask. Thank you.  At this time do you have any question to ask me about this survey before we begin? 1= Yes 2=No  Do I have your consent to begin the interview now? 1=Yes 2=No  Signature of the interviewee ………………………………………….. | | |
| --- | --- | --- |
| **Section B: Demographic Information (circle the appropriate code)** | | |
| No | Questions and filters | Coding categories |
| 1.1 | Age of the respondent in years?  *If given age is doubtful*, *ask for date of birth then calculate the age.* | 1. 30-39 2. 40-49 3. 50-59 4. 60-69 5. Other (please specify)--------- |
| 1.2 | What is the highest level of school you attended?  Educational level? | 1. Never went to school 2. Primary 3. Secondary 4. Tertiary level |
|  | What is the highest grade/form/year you completed at that level? | --------------------- |
| 1.3 | Religious affiliation? | 1. SDA 2. AIC 3. Catholic 4. Other (please specify)----------- |
| 1.4 | Occupation? | 1. House wife 2. Business 3. Farming 4. Formal employment (specify)--- 5. Other (specify)….. |
| 1.5 | What is your marital status? | 1. Never married 2. Married 3. Separated 4. Widow 5. Other (please specify)---------- |
| 1.6 | Tribal affiliation by marriage | ______ |
| 1.7 | Tribal affiliation by birth | _______ |
| 1.8 | Where do you live?  **Probe:** | 1. At the nearby shopping centre 2. In the rural home 3. Other (specify)------- |
| 1.9 | Whom do you stay with? **Probe** | ___________ |
| 1.10 | Do you have a mother in-law (If not married mother)? | 1. Yes 2. No |
| 1.11 | Does she stay near you? | 1. Yes 2. No |
| 1.12 | Have you ever stayed anywhere else? | 1. Yes 2. No |
| 1.13 | If yes where? **Probe** | ---------- |

**PART C: FOOD TABOOS TO BE AVOIDED**

*Ok, great! Now I’m going to ask you some questions specifically about what women eat or avoid while they are pregnant.*

- 1. According to the Nandi (if the respondent is not Nandi, otherwise replace) cultural beliefs, which foods and drinks are not supposed to be consumed by pregnant women?

*In case of a no response in the last column, ask: if you are given will you eat it? Kindly probe*

| **Fruits and Vegetables** | | Reason for avoiding it? **Probe:** | Do you eat it? Yes/no, **probe:** |
| --- | --- | --- | --- |
| Local name | English Name |  |  |
|  |  |  |  |
|  |  |  |  |
|  |  |  |  |
|  |  |  |  |
|  |  |  |  |

| **Animals and Animal Products** | | Reason for avoiding it? Probe: | Do you eat it? Yes/no, **probe:** |
| --- | --- | --- | --- |
| Local name | English name |  |  |
|  |  |  |  |
|  |  |  |  |
|  |  |  |  |
|  |  |  |  |
|  |  |  |  |
|  |  |  |  |
|  |  |  |  |

| **Drinks and beverages** | | Reason for avoiding it | Do you drink it? Yes/no, **probe:** |
| --- | --- | --- | --- |
| Local name | English name |  |  |
|  |  |  |  |
|  |  |  |  |
|  |  |  |  |
|  |  |  |  |
|  |  |  |  |

| **Drugs and medicines**  ***Both local and hospital drugs*** | | Reason for avoiding it | Do you take it? Yes/no, **probe:** |
| --- | --- | --- | --- |
| Local name | English name |  |  |
|  |  |  |  |
|  |  |  |  |
|  |  |  |  |
|  |  |  |  |

**PART E: CULTURALLY IMPOSED FOOD PREFERENCES**

3.1 According to the Nandi(if not Nandi, otherwise replace) cultural beliefs, which foods and drinks highly recommended for pregnant women? *For No responses in the last column, ask, in case of drought and famine, will you eat them?*

| **Fruits and Vegetables** | | Reason for avoiding it? **Probe:** | Do you eat it? Yes/no, **probe:** |
| --- | --- | --- | --- |
| Local name | English Name |  |  |
|  |  |  |  |
|  |  |  |  |
|  |  |  |  |
|  |  |  |  |
|  |  |  |  |

| **Animals and Animal Products** | | Reason for avoiding it? Probe: | Do you eat it? Yes/no, **probe:** |
| --- | --- | --- | --- |
| Local name | English name |  |  |
|  |  |  |  |
|  |  |  |  |
|  |  |  |  |
|  |  |  |  |
|  |  |  |  |
|  |  |  |  |
|  |  |  |  |

| **Drinks and beverages** | | Reason for avoiding it | Do you drink it? Yes/no, **probe:** |
| --- | --- | --- | --- |
| Local name | English name |  |  |
|  |  |  |  |
|  |  |  |  |
|  |  |  |  |
|  |  |  |  |
|  |  |  |  |

| **Drugs and medicines**  ***both local and hospital drugs*** | | Reason for avoiding it | Do you take it? Yes/no, **probe:** |
| --- | --- | --- | --- |
| Local name | English name |  |  |
|  |  |  |  |
|  |  |  |  |
|  |  |  |  |
|  |  |  |  |

**PART F: EVENTS AND ACTIVITIES TO DO/AVOID**

4.1 Other than foods, are there other things/activities a pregnant woman is not supposed to do? *(Try to establish the one that impacts on nutritional intake and in case of a no answer in the last column, probe)*

| **Events/activities to avoid** | | Reason for discouraging it | Do you avoid them? Yes/no |
| --- | --- | --- | --- |
| Local name | English name |  |  |
|  |  |  |  |
|  |  |  |  |
|  |  |  |  |
|  |  |  |  |
| **Events/activities to do** | | Reason for encouraging it | Do you practice them? Yes/no |
| Local name | English name |  |  |
|  |  |  |  |
|  |  |  |  |
|  |  |  |  |
|  |  |  |  |

**PART G: POSIBILITY OF CHANGE IN THESE FOOD BELIEFS**

| No | Questions and filters | Coding categories**(circle the appropriate code)** |
| --- | --- | --- |
| 4.1 | Has the food beliefs mentioned above changed over time? | 1. Yes 2. No |
| 4.2 | If yes please tell me how |  |
| 4.3 (a)  (b)  (c)  (d) | In this community Is there a tendency or trend in lessening or abandoning certain rules of food beliefs? | 1. Yes 2. No |
|  | If yes, please give me examples of food beliefs that have been abandoned so far |  |
|  | Tell me the reasons why they have been abandoned |  |
|  | If no, what will happen if someone abandons a food belief? |  |
| 4.5 | What is your own opinion about the tabooed foods and drinks during pregnancy? |  |
| 4.6 | What is your own opinion about the culturally recommended foods and drinks during pregnancy? |  |

**PART H: 24 HOUR DIET RECALL AND CULTURAL FOOD PRACTICES:**

| 5.1 | What did you eat in the? |  |
| --- | --- | --- |
|  | Morning |  |
|  | Mid-day |  |
|  | Evening |  |
|  | Between the meals |  |
| 5.2 | Does what you ate differ from what others ate? |  |
| 5.3 | If yes how? |  |
| 5.4 | Who generally decides what the family eats in this community? |  |
| 5.5  a)  b)  c)  d) | How is food distributed in your community? In terms of: |  |
|  | Sitting order? |  |
|  | Who eats first? (Men eat first; children eat first, women eat “leftovers,” etc.) |  |
|  | Do members of the household eat from the same plate or from an own plate? |  |
|  | Does this change when there is a pregnant woman in the family (do they become prioritized or not)? *Probe on what priorities are they given?* |  |
| 5.6 | What kinds of foods are consumed during food shortages and famine by pregnant women? |  |
| 5.7 | Are pregnant women given special consideration during drought and famine? |  |
| 5.8 | What kinds of commodity, both food and non-food, would pregnant women like to buy when sufficient money is available? |  |
| 5.9(a)  (b)  (c)  (d) | Are there periods of fasting in a year in this community? |  |
|  | Is this a community event or done on an individual basis? |  |
|  | How do you fast? |  |
|  | Do pregnant women fast as well or are they exempted from fasting? |  |

**PART I: KNOWLEDGE ON NUTRITION AND INTERVENTION PROGRAMS**

| 6.1 | Who generally advises pregnant women on what they should eat at home in this community? |  |
| --- | --- | --- |
| 6.2 | Other than the person mentioned in 5.1, who else advices pregnant women on what they should eat? |  |
| 6.3a)  b)  c)  d)  e) | Are you able to recognize signs and symptoms of a poorly nourished pregnant woman? | 1. Yes 2. No |
|  | If yes, can you tell me some signs and symptoms of a poorly nourished person? (probe on deficiencies such as vitamin A, iodine or iron) |  |
|  | Are you able to relate these signs and symptoms with nutritional deficiencies such as iron, iodine, vitamin A? | 1. Yes 2. No (if yes probe) |
|  | Are people worried about these signs and symptoms or they regard them as normal? | 1. yes 2. no |
|  | What kind of actions and measures are taken to reduce these signs and symptoms? |  |
| 6.4 a)  b)  c)  d) | Is there any health problem associated with malnutrition during pregnancy? | 1. Yes 2. No |
|  | If yes, how does it affect the mother? |  |
|  | If yes, how does it affect the child? (in terms of birth weight and child’s health, etc) |  |
|  | (If the respondent has some knowledge on nutrition, ask) where did you receive this information from? |  |
| 6.5 a)  b)  c) | Other than your friends and relatives, has someone else ever advised you on how you should eat? | 1. Yes 2. No |
|  | If yes, who informed you? |  |
|  | If yes, what information did you receive? |  |
| 6.6 | Which other structures exist in the community to support mothers on healthy eating and/ or crop production? |  |
| 6.7  a)  b)  c) | How best should messages on maternal nutrition be communicated to the community? i.e |  |
|  | Where the messages should be communicated? |  |
|  | Who should pass the messages? |  |
|  | Which media should be used? |  |
| 6.8 | How can mothers be supported in order to eat well? |  |
| 6.9 | Who should be members of mother support groups? |  |
| 6.10(a  b)  c)  d) | What are your views about the role of nurses in promoting nutritional advice in this community? | 1. They do it well 2. They are not doing it well |
|  | What do you think are some of the things that are working well? Why? |  |
|  | What do you think are some of the things that are not working well? |  |
|  | What do you think can be done to improve their work? |  |

**Conclusion:**

4.12. What are some recommendations you have to improve the health of mothers?

4.13. Do you have any questions for us?

Thank you for your time today. We greatly appreciate you coming to talk with us today.

**INTERVIEWER ASSESSMENT**

Interviewer, please complete the questions below based on your own observation and assessment of the entire interview process and of the respondent

1. What is your assessment of the respondent's concentration and attentiveness during the interview?

1=Very good 2=Good 3=Moderate 4=Bad 5=Very bad

2. What is your evaluation of the accuracy and completeness of the respondent's answers?

1=Very high 2=High 3=Average 4=Low 5=Very low

3. What is your assessment of the respondent's comprehension of issues discussed?

1=Very good 2=Good 3=Moderate 4=Bad 5=Very bad

**Interviewer notes**
